# Unmasking Depression in Dementia: A Comprehensive Review of Symptom Overlap and Management

**DOI:** 10.1192/j.eurpsy.2025.664

**Published:** 2025-08-26

**Authors:** A. H. I. Abu Shehab, A. Baltă, A. B. Ciubară, S. L. Burlea, V. Doina Carina, M. Grigoraș, A. Ciubară

**Affiliations:** 1 Psychiatry, “Elisabeta Doamna” Psychiatry Hospital of Galati; 2 Clinical; 3 orthopaedics and traumatology, Faculty of Medicine and Pharmacy, “Dunărea de Jos” University, Galati; 4 Oral and maxillofacial surgery, 7University of Medicine and Pharmacy “Grigore T. Popa”, Iași; 5 Rheumatology; 6 Psychology; 7 Psychiatry, Faculty of Medicine and Pharmacy, “Dunărea de Jos” University, Galati, Romania

## Abstract

**Introduction:**

Depression is a prevalent yet frequently underdiagnosed condition in patients with dementia, exacerbating cognitive decline and emotional health. The combination of depressive symptoms with dementia-related cognitive deficits complicates accurate diagnosis and management of depression in this group of patients. Improved diagnostic instruments specifically designed for dementia patients are essential for improving detection and treatment outcomes.

**Objectives:**

To evaluate the prevalence and diagnostic challenges of depression in dementia, focusing on symptom overlap. Additionally, it aims to assess the efficacy of diagnostic tools and management strategies to improve patient outcomes.

**Methods:**

A systematic review of the literature was performed to evaluate the prevalence, symptomatology, and diagnostic difficulties of depression in dementia.

**Results:**

The results reveal a substantial intersection between the cognitive and emotional symptoms of dementia and depression, encompassing apathy, anhedonia, and mood disorders. The coexistence of these symptoms frequently results in the underrecognition of depression or its misattribution to the dementia process. Instruments such as the CSDD and NPI showed higher sensitivity in identifying depression among dementia patients. Pharmacological therapies, including psychotherapy and individualized behavioral interventions, demonstrated improvements in mood and cognitive function; nevertheless, therapeutic success fluctuated according to the stage of dementia.

**Conclusions:**

Depression in dementia patients has distinct diagnostic and treatment issues owing to symptom overlap and cognitive deterioration. Enhanced diagnostic instruments, including the CSDD, NPI, and GDS, provide superior distinction of depression symptoms from dementia-related deficits. Early recognition and tailored treatment strategies, integrating pharmaceutical and behavioral interventions, may alleviate depression symptoms and enhance overall patient outcomes. Interdisciplinary collaboration is crucial for enhancing care.

**Disclosure of Interest:**

None Declared

